# The Role of NADPH Oxidase 2 in Leukocytes

**DOI:** 10.3390/antiox14030309

**Published:** 2025-03-04

**Authors:** Jennifer W. Leiding, Clayton E. Mathews, Danielle E. Arnold, Jing Chen

**Affiliations:** 1 Division of Allergy and Immunology, John Hopkins University, Baltimore, MD 21218, USA; jleidin1@jhmi.edu; 2 Cancer and Blood Disorders Institute, Johns Hopkins All Children’s Hospital, St. Petersburg, FL 33701, USA; 3 Department of Pathology, Immunology and Laboratory Medicine, College of Medicine, University of Florida, Gainesville, FL 32610, USA; cxm@ufl.edu; 4 Department of Infectious Diseases and Immunology, College of Veterinary Medicine, University of Florida, Gainesville, FL 32610, USA; 5 Immune Deficiency Cellular Therapy Program, National Cancer Institutes, National Institutes of Health, Bethesda, MD 20892, USA; danielle.arnold@nih.gov

**Keywords:** NADPH oxidase, NOX2, reactive oxygen species, leukocytes, chronic granulomatous disease, autoimmunity

## Abstract

NADPH oxidase (NOX) family members are major resources of intracellular reactive oxygen species (ROS). In the immune system, ROS derived from phagocytic NOX (NOX2) participate in both pathogen clearance and signaling transduction. The role of NOX2 in neutrophils and macrophages has been well studied as mutations in NOX2 subunits cause chronic granulomas disease (CGD). NOX2 is expressed across a wide range of immune cells and recent reports have demonstrated that NOX2-derived ROS play important roles in other immune cells during an immune response. In this review, we summarize current knowledge of functions of NADPH oxidase 2 in each subset of leukocytes, as well as associations of NOX2 deficiency with diseases associated specifically with autoimmunity and immune deficiency. We also discuss important knowledge gaps as well as potential future directions for NOX2 research.

## 1. Introduction

Leukocytes, also known as white blood cells, are immune cells that are made in bone marrow. These cells circulate in the blood stream and lymphatic system and are also found in the spleen, lymph nodes, and other secondary lymphoid structures. Leukocytes include granulocytes (neutrophils, eosinophils and basophils), monocytes and lymphocytes (T and B cells). Each type of leukocyte plays an important and distinct role in keeping immune homeostasis, defending against infections and destroying malignances. NADPH oxidase (NOX) is a family of enzymes found in most nucleated human cells. NOXs are major sources of cellular reactive oxygen species (ROS). In the human body, ROS are involved in many physiological and pathophysiological processes. Excessive ROS cause cell and tissue damage and lead to diseases, while complete lack of ROS results in immunodeficiency and dysregulated signaling pathways. In the immune system, ROS directly participate in pathogen clearance, and mediate signal transduction during immune responses. Here, we review the current understanding of NOX-derived ROS in each type of leukocytes, diseases associated with NOX deficiency, and other defects that result in ROS dysregulation. Since the role of NOX in phagocytes is broadly studied and well understood, here we emphasize NOX2 in non-phagocytic leukocytes, T cells, B cells and NK cells. We focus on the association between NOX2 deficiency and autoimmune diseases, and discuss the role of NOX2 in lymphocytes during the pathogenesis of autoimmunity. Finally, we discuss gaps in knowledge and the needs of future investigations in this field to fully elucidate the role of this essential family of enzymes.

## 2. NADPH Oxidase Isoforms and Distribution

Nicotinamide adenine dinucleotide phosphate (NADPH) oxidase (NOX) is a family of enzymes found in most nucleated cells in the human body. These enzymes oxidize NADPH to form NADP and free radicals, and therefore are a major source of intracellular free radical production. The NADPH oxidase is comprised of six proteins; gp91^phox^ and p22^phox^, encoded by *CYBB* and *CYBA,* respectively, are expressed on the cell membrane, while p47^phox^, encoded by *NCF1*, p67^phox^ encoded by *NCF2*, and p40^phox^ encoded by *NCF4,* are cytoplasmic. Upon activation, cytoplasmic proteins co-localize and migrate to the membrane, where they interact with membrane subunits and the small GTPases (Rac1/Rac2) to form the active NOX2 complex. Electron transfer from NADPH to molecular oxygen then occurs resulting in production of a superoxide anion (O_2_^−^). Superoxide is then converted to hydrogen peroxide (H_2_O_2_) by superoxide dismutase and H_2_O_2_ is converted to hypochlorous acid (HOCl) by myeloperoxidase, which has direct killing effects on microbes. Further, the negative charge created by superoxide anions intracellularly elicits potassium (K^+^) influx that also enhances cellular killing [1]. Development of neutrophil extracellular traps (NETs) are also dependent on ROS. NETs are extracellular decondensed nuclear chromatin released by neutrophils to kill microbes and are therefore also defective in CGD due to inadequate ROS production.

**Figure 1:**
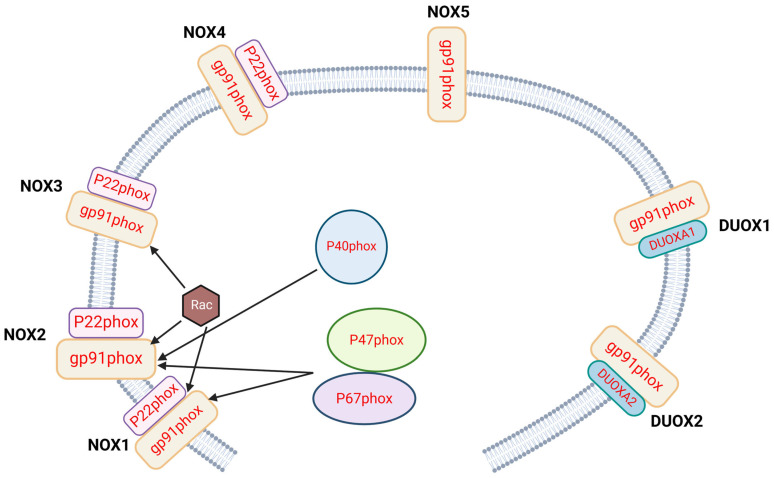
Seven isoforms of NADPH oxidase and their subunits. gp91^phox^ is the common catalytic core for each isoform. Another membrane subunit P22^phox^, is common to NOX1, NOX2, NOX3 and NOX4. NOX1 and NOX2 recruit the cytoplasmic subunit P47^phox^, Rac1 and P67^phox^ upon activation to form functional complexes; NOX2 also recruits the P40^phox^ subunit. NOX5 does not have the membrane subunit P22^phox^, and does not recruit the cytoplasmic subunit to form multimers like the other NOX isoforms. Likewise, DUOX1 and DUOX2 isoforms do not have the membrane subunit P22^phox^, rather, these isoforms have DUOXA1 and DUOXA2, respectively. Arrows indicate the recruitment of intracellular subunits to the cytoplasmic membrane upon activation.

There are seven isoforms of NOX in humans: NOX1, NOX2, NOX3, NOX4, NOX5 DUOX1 and DUOX2. The composition and assembly mechanism of subunits in each isoform are well summarized by Cipriano et al. in these recent reviews [2,3]. Figure 1 shows seven isoforms of NOX and their subunits. Briefly, NOX are widely expressed by many cell types, including immune cells. NOX 1 is distributed in the colon [4], prostate [5], placenta [6], and vascular smooth muscle cells [7]; NOX 2 was first discovered in phagocytes (discussed in Section 3 below); NOX3 is expressed in the inner ear [8]; NOX4 is found in a wide variety of tissues, including thyroid [9], blood vessel and heart [10], kidney [11], lung epithelial cells [12], chondrocytes [13], and tumors [14]. NOX5 is also widely distributed in different tissues and cell types [15]. DUOX1 is mostly distributed in the thyroid [16] and respiratory system [17]; DUOX2 is found in the thyroid [18], digestive system [19], and like many isoforms, is associated with cancer. The review by Nocella et al. [3] thoroughly discussed CGD as well as pathologies associated with hyperactivation of NOX, with the focus being on the latter. Therefore, our review, on the other hand, focuses on NADPH oxidase in each subset of non-phagocytic leukocytes, and in-depth clinical features of CGD, especially the autoinflammation and autoimmunity observed in these patients.

## 3. Role of NOX in Neutrophils

NOX2 in neutrophils was the first NOX isoform discovered [20,21], and therefore it was named phagocytic NOX. To date, neutrophil NOX2 is the most thoroughly studied NOX isoform. Neutrophils play a key role in pathogen elimination in the innate immune system. NOX2 in neutrophils is the major source of ROS production. Once neutrophils endocytose pathogens, neutrophil NOX is activated and the proper amount of ROS are generated to destroy pathogens. The role of NOX2 in neutrophils is well understood and is already dogma. Signaling pathways involved in neutrophil NOX2 activation are well reviewed by Belambri et al. [22]. Clinical manifestations of NOX deficiency are considered to be mostly due to NOX2 deficiency in neutrophils (discussed in detail in Section 6 below).

## 4. NOX in Lymphoid Cells

Lymphoid cells are the main cell types of the immune system. Lymphoid cells include T cells, B cells and Natural Killer (NK) cells. Lymphoid cells originate from bone marrow. T cells further maturate in the thymus, while B cells and NK cells mature in the bone marrow and circulate to peripheral lymphoid tissues. These cells eliminate pathogens and infected, damaged or malignant cells.

### 4.1. NOX and T Cells

T cells, together with B cells, are the main component of the adaptive immune system. They belong to lymphocytes. These cells are essential for immune response to bacterial, fungal and viral infections, and tumor surveillance. Proper T cell immune responses relay on appropriate signaling and balanced regulation of T cell subsets. Each subtype of T cells, such as CD8^+^, CD4^+^, and regulatory T cells (T regs), plays distinct yet interrelated roles in response to infection and tumor development. CD8^+^ T cells are considered cytotoxic and directly kill infected cells or tumor cells, while CD4^+^ T cells function through coordination with other immune cells, such as B cells, macrophages, and dendritic cells, which all belong to antigen presenting cells (APC), to carry out anti-infection and anti-tumor tasks. T regs regulate the balance in the immune system by suppressing over activations and autoimmunity. The balance among these subsets determines a normal immune response and keeps homeostasis. The role of NOX2 in phagocytes are well understood as discussed in Section 3 of this review, while involvement of NOX in T cell signaling are less explored and have not drawn attention until recent years. Yet, ROS as a signal in physiological and pathological processes have been long recognized. T cells lack proper ROS signaling, perform poorly in the immune response, and result in either immune deficiency or autoimmunity [23,24,25].

**Figure 2:**
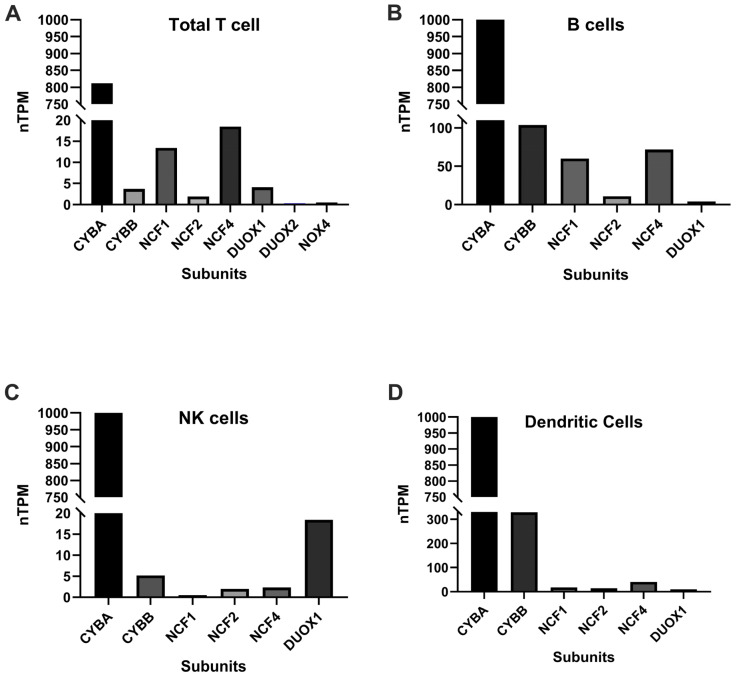
NOX subunits are expressed at different levels by human T cells (**A**), B cells (**B**), NK cells (**C**) and dendritic cells (**D**). Single cell RNA data from Human Protein Atlas (https://www.proteinatlas.org/ (accessed on 2 October 2024)). nPTM: normalized transcripts per million.

The expression of NOX by T cells has been noticed for 20 years [26]. The fact that T cells express NOX2 has been shown at both the protein and mRNA levels. Originally, using an antibody (clone 7D5), the subtype of NOX expressed by T cells was thought to be the phagocyte type [26]. In fact, several reports have observed both human and mouse T cell expression of phagocyte type NOX2, including subunits p47^phox^, p67^phox^ and gp91^phox^ [26,27]. Additionally, single cell RNA sequencing (scRNA-seq) data indicate the genes encoding these NOX subunits, *CYBA* (p22^phox^), *NCF4* (p40^phox^), *NCF1* (p47^phox^), *CYBB* (gp91^phox^), and *NCF2* (p67^phox^) are expressed in human T cells (Figure 2A). Regarding roles of NOX2 in T cells, early studies show elimination of NOX2 in CD8^+^ T cells modulates JNK and NFκB signaling, with decreases in both IFNγ levels and CD39 expression [28], indicating NOX2-derived ROS control Tc1 differentiation. T cell NOX is activated upon TCR stimulation [26], suggesting role of NOX ROS in TCR signaling. Non-phagocytic NOX, Duox1, has also been reported to be involved in the TCR signaling cascade, including phosphorylation of ZAP70, calcium flux and cytokine production [29]. NOX2 transfers from CD8^+^ T reg (CCR7^+^) to CD4^+^ T cells suppressing CD4^+^ activation by anti-CD3 [30].

**Figure 3:**
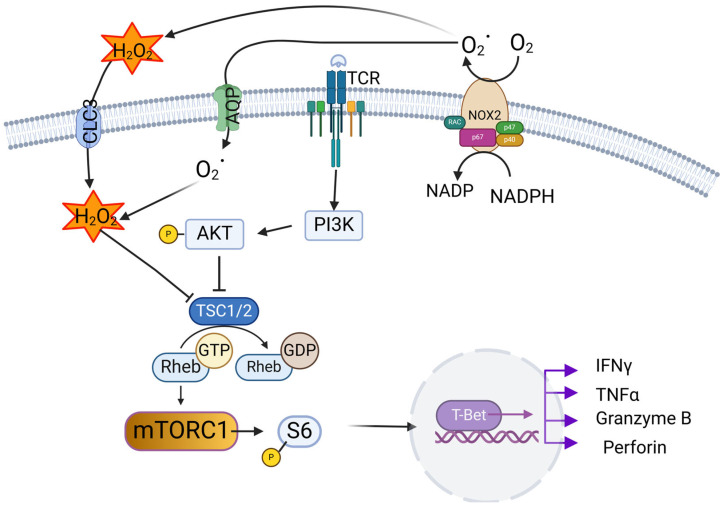
NOX2-ROS regulate CD8^+^ T cell activation and effector function through the mTOR pathway in mouse and human NOX2-deficient T cells. ROS produced by NOX2 are transported inside of cells by Aquaporin (AQP) or CLC3. Upon TCR stimulation, AKT is phosphorylated by PI3K. Phosphorylated AKT further phosphorylates multiple sites on TSC2, which forms a functional complex with TSC1. Phosphorylation of TSC2 impairs the ability of the TSC1/2 complex to act as a GTPase-activating protein toward the small GTPase Rheb, allowing Rheb-GTP to accumulate. Intracellular ROS facilitate the inhibition of TSC1/2, resulting in reduced conversion of Rheb-GTP to Rheb-GDP. Increased Rheb-GTP activate mTORC1 and leads to translation of genes encoding effector molecules.

Our studies indicated that mouse models of NOX deficiency in CD8^+^ T cells demonstrated decreased cytotoxic function and in an autoimmune model failed to induce the autoimmunity [24,31]. Interestingly, a lack of NOX ROS in CD4^+^ T cells results in accelerated autoimmune diabetes [32], suggesting the distinct role of NOX in each T cell subtype and the complicated interaction between T cells and non-T cell compartments in the immune system, such as macrophages and B cells [33]. Patients that carry mutations in genes encoding NOX subunits develop chronic granulomatous disease (CGD, detailed below in Section 6 of this review). A few observations regarding T cell dysfunction in CGD have been described, including CD4^+^ T cell reduction, skewing to a Th17 phenotype with an increase in IL-17 and TNFα [34], and higher activation markers in Vδ2 within CD8^+^ T cells [35,36]. Consistently, studies (ours and others) using animal models have suggested abnormalities in the T cell compartment associated with NOX2-deficiency. T cells from CGD patients and mice with a deletion of *Ncf1* exhibit a Type 1 interferon gene expression signature [37]. Our study indicates NOX2-deficient human and mouse CD8^+^ T cells produce lower levels of IFNγ and TNFα upon activation [24]. CD4^+^ T cells from X-CGD female carriers have also shown lower IFNγ production after being stimulated overnight with IL-2 plus PMA [38]. Interestingly, mouse CD4^+^ T cells show a different response. Purified CD4^+^ T cells deficient of gp91^phox^ from C57BL/6 mice show increased Th1 cytokine secretion upon TCR stimulation [5]. Likewise, CD4^+^ T cells from a mouse strain carrying diabetogenic TCR, NOD.BDC2.5 mice, become more diabetogenic when they carry a mutation in the *Ncf1* gene and display increased Th1 cytokine secretion [32]. Different responses to NOX2 deficiency suggest either NOX2-ROS control CD8^+^ and CD4^+^ T cell subsets through distinct pathways, or an interaction of NOX2-ROS from other immune cells and T cells since contrasting results were observed when using bulk samples versus purified CD4^+^ or CD8^+^ T cells. Nonetheless, these studies suggest regulatory roles of NOX2-ROS in T cell differentiation and signaling. Mitochondria are another source of cellular ROS. The role of NOX2-ROS as compared to mitochondrial ROS in T cell signaling is poorly understood. Mitochondrial ROS are thought to be indispensable for Th17 differentiation in a mouse model of autoimmune arthritis [39], while NOX2-ROS are likely to promote Tc1 and Th1 development. We also found that NOD, a mouse model of Type 1 Diabetes, with a non-functional *Ncf1* are prone to EAE induction [25]. But the pathophysiology of T cell dysregulation in a natural NOX2-ROS-deficient environment in CGD and the mechanism and pathophysiology of autoimmunity and autoinflammation in CGD remain unknown. In addition to CD4^+^ T cell skewing to a Th17 phenotype, abnormal T regs have been implicated in the higher incidence of autoimmunity and autoinflammation in CGD patients, but only minor differences were detected in T regs from CGD patients and controls [40]. Possible reasons for conflicting results might be experiments using bulk cells versus isolated cells, residual NOX function in patients vs. complete inhibition with chemical inhibitors in experimental settings, and animal models on different genetic background, such as non-autoimmune C57BL/6 vs. autoimmune NOD. These prior studies have indicated NOX2-ROS from other cell types participate in T cell activity; NOX2-ROS from activated neutrophils have been shown to cause structural modification of proximal TCR components [41]. However, modifications of other proteins and molecules along the TCR signaling pathway have not been explored. This is partially due to a historical lack of technology for detecting large-scale post-translational modification events, such as phosphorylation. Neutrophils act as negative modulators of psoriasis propagation and exacerbation by inhibiting γδ T cell effector functions via NOX-ROS production [42]. A balance between NOX2-ROS from CD4^+^ T cells and from macrophages controls proper differentiation of Th1 [25]. NOX also controls the mouse MHC class II repertoire [43]. These results clearly show that NOX2-ROS are involved in T cell signaling. In our recent publication [24], we postulated that NOX2-ROS regulate CD8^+^ T cell activation and effector function through the mTOR pathway in mouse and human NOX2-deficient T cells. We concluded that NOX2-ROS play a non-redundant role in the TCR-mediated CD8^+^ T cell effector function (Figure 3).

Regulatory T cells (T regs) are a subset of CD4^+^ T cells that are essential in keeping immune balance by suppressing overreactive immune responses. As a subset of CD4^+^ T cells, T regs interact closely with other immune cells, such as macrophages and dendritic cells. T reg deficiency results in autoimmunity [44]. NOX-derived ROS from macrophages are thought to induce T regs [45]. Evidence indicates that NOX deficiency in T regs leads to imbalanced immunity. T regs from patients with liver cirrhosis exhibit enhanced NOX2 activity together with mitochondrial abnormalities that result in increased ROS, defective T reg function and lower viability [46]. T reg percentages are increased in X-CGD patients [47]; however, abnormal T regs have been implicated but only minor differences were detected in T regs from CGD patients and controls [40] that cannot explain the increased autoimmunity in these patients. In NOD mice CD4^+^ T cells with NOX2 deficiency are more diabetogenic, and T regs function is impaired [32]. The above-mentioned observation that T reg deficiency in different animal models leads to different results, either enhancing or reducing autoimmunity, suggests that there is a distinct regulatory role of NOX-ROS in different cell subsets.

Invariant Natural Killer (iNKT) cells are a special type of T cell that express an invariant TCR and a number of cell surface molecules in common with natural killer (NK) cells [48]. These cells regulate other immune cells through cytokines. In an animal model of Ischemia–Reperfusion lung injury [49], P47^-/-^ or NOX2^-/-^ iNKT cells adoptively transferred to iNKT cell-deficient mice that went through Ischemia–Reperfusion-attenuated lung injury and IL-17 production, suggesting that NOX2 plays a key role in inducing iNKT cell-mediated IL-17 production and subsequent lung injury after Ischemia–Reperfusion [49].

### 4.2. NOX and B Cells

B lymphocytes are essential cells within the adaptive immune system. B cell subsets in the peripheral blood start at the transitional B cells, recent bone marrow emigrants that are the most immature in development stage. These cells differentiate through the T1, T2, and T3 stages into naïve B cells. Naïve B cells have not yet encountered antigen and have not undergone maturation in spleen, lymph node, or other lymphoid tissue. Upon an antigen encounter, naïve B cells differentiate through the aNAV stage to one of three major groups of B cells. The majority of B cells belong to the conventional B2 cells. Additionally, there are rare populations of B1 (phagocytic B cells) and B reg (Regulatory B cells). The development and surface markers of B cell subgroups are well summarized by Ichwaku et al. in a recent review [50].

B cell receptors (BCR: antibody bound to a Fc receptor) on the surface of B cells allow antigen recognition and activation. Conventional B2 cells are the major players in humoral immunity. Once the BCR is stimulated by antigen, B2 cells are activated and produce antibodies to fight against infection in an antigen-specific fashion. Based on single cell RNA seq data, B cells express several NOX subtypes, with *CYBA* being highly expressed followed by *CYBB* (Figure 2B). BCR stimulation induces ROS production from NOX2, which is thought to promote B cell activation and proliferation [51]. The role of ROS as a second messenger in BCR signaling was recognized in the 1990s [52], when cells were transfected to express BCR and treated with pervanadate/H_2_O_2_, phosphorylation events were induced downstream of BCR with a pattern that was similar to what happens when BCR is stimulated with antigen [52]. BCR engagement evokes a series of intracellular events, including activation or inhibition of kinases, phosphatases, and protein ubiquitination (reviewed in [53]). BCR stimulation induced a two-phase ROS release [51]. The first phase is regulated by NOX2 and occurs within minutes to 4 h after BCR ligation [54]. The second phase results from mitochondrial ROS (mtROS) and is prolonged (6–24 h) [51]. The role of ROS, especially NOX2-derived ROS in B cell signaling has been well summarized in this recent review [55]. NOX2-derived H_2_O_2_ is transported by aquaporin 8 across the plasma membrane to regulate tyrosine phosphatases and kinases [56]. NOX2-ROS increase tyrosine phosphorylation in B and T cells [57]. BCR-dependent NOX2 activity relies on spleen tyrosine kinase (SYK), bruton tyrosine kinase (BTK), and phosphatidylinositol-specific phospholipase Cγ2 (PLCγ2), to regulate calcium mobilization downstream of BCR ligation [58]. NOX2-ROS regulate BCR signaling through reversibly oxidizing signaling molecules, including protein tyrosine phosphatases, such as protein tyrosine phosphatase 1B (PTP1B), SHP2, and PTEN at the catalytic cysteine residue [54]. These oxidation events inhibit phosphatase activity to prolong signaling phosphorylation events and enhance cell signaling. ROS also reversibly oxidize kinases, such as Src family kinases, and transcriptional factors, such as NF-κB and p53 [54]. Inhibition of the protein tyrosine kinases activity or the PI3K activity, or depletion of Ca2^+^ led to a significant reduction in anti-IgG-stimulated ROS production in B cells [59]. While deletion of NOX2 promoted antigen-specific BCR stimulation-induced proliferation [58], suggesting NOX2-ROS are necessary regulators for proper B cell activation upon BCR ligation that is not overreacted or underreacted. In addition to producing antibodies, B cells also serve as antigen-presenting cells. NOX-ROS regulate B cell antigen-presenting functions by altering the repertoire presented by MHC class II to CD4^+^ T cells [60]. NADPH oxidase and p40^phox^ play a role in skewing B cell epitope selection and T cell recognition of self-antigen [61].

The phagocytic B1 cells [62,63,64] also utilize NOX2-ROS. These phagocytic B1 cells are identified by markers B220^low^, CD19^+^,CD11b^+^. B1 cells include two subgroups: B1a (CD5^-^) and B1b (CD5^+^) [50,64]. These cells are stimulated by T cell-independent antigens and produce antigen non-specific “natural antibodies” IgM and IgD, and cytokines that are immunoregulatory [65], as reviewed in [66]. B1 cells are sensitive to ROS, as B1 but not conventional B2 cells with deficient glutathione peroxidase experience abnormal antibody response, increased lipid peroxidation, and ferroptosis [67]. The sensitivity is due to redox-sensitive organelle peroxisome [68]. These B1 cells engulf pathogens and this phagocytic ability is mediated by NOX2-derived ROS [69].

Regulatory B cells (B regs) are a group of immunosuppressive cells that are important for balanced immune reactions and immune homeostasis [70]. B regs suppress inflammatory lymphocytes, such as T cells, by secreting regulatory cytokines IL-10, IL-35, and TGF-β [71]. Although there are no definitive makers to distinguish B regs, IL-10^+^ B cells are generally considered B regs; therefore, B regs and B2 cells are not mutually-exclusive [71]. B regs can be induced by proinflammatory cytokines, TLR ligands, B cell-activating factor (BAFF), and A proliferation-inducing ligand (APRIL). NOX2-ROS deficiency amplified TLR4 signaling in *Nrf2*^-/-^ mouse macrophages [72]. Mouse and human B cells deficient of NOX2 also showed enhanced TLR7 signaling [73]. Antioxidant N-acetyl-L-cysteine (NAC)-treated or peroxiredoxin II (PrxII)-deficient mouse spleen cells showed inhibited BAFF expression [74], suggesting ROS play a role in the regulation of BAFF, although the authors did not explore whether the regulatory ROS is NOX derived. Collectively, these data suggest NOX2-ROS have a role in the induction of B regs.

NOX2 in B cells are also involved in autoimmunity. A recent study using SLE mouse models, Liu et al. [73], showed *Ncf1*^-/-^ B cells on the C57BL/6 genetic background exhibited competitive advantage in germinal center (GC) entry, expansion/selection, enhanced humoral responses to TLR-containing viral antigens, and increased class-switched IgM- IgD- B cells. The authors also showed that enhanced TLR signaling was due to dysregulation of endolysosome trafficking [73]. Another study using SLE mouse models showed that B cell-specific deletion of *Cybb* increased kidney pathology and altered antibody responses, and these changes are TLR7 dependent [75], suggesting a protective role of B cell NOX2 in the SLE model through regulation of TLR7. In a collagen-induced arthritis mouse model on a C57BL/10 background [76], the authors observed that a mutation in the lipid endosomal membrane binding site (R58A) of the NCF4 subunit resulted in reduced intracellular ROS production in B cells after stimulation, increased severity of arthritis symptoms, with enhanced plasma cell formation, and increased antibody production. These changes were linked to increased CXCR3 and decreased CXCR4 expression. B10.Q mice with a mutated and non-functional *Ncf1* showed enhanced autoimmunity (collagen-induced arthritis and experimental allergic encephalomyelitis) [77]. Although these are T cell-mediated autoimmune diseases, the levels of antibodies to collagen were higher in *Ncf1* mutant mice, and the effect most pronounced for T cell-dependent production of IgG and not IgM [77]. Autoantibodies associated with Crohn’s disease are present in CGD patients regardless of colitis [78]. CGD patients and mouse models showed increased IgG2 subtype compared to controls, while IgG1 levels were lower in patients than controls, and higher in mutant mice [79]. The authors suggested that enhanced Th1 response led to deviation toward IgG2c responses [79]. BCR-induced ROS in B cells negatively regulated T cell-independent Type 1 antibody response [58]. Non-immunized gp91^phox^ KO mice showed enhanced serum titers of IgG1 but not of IgM, IgG3, IgG2a and IgG2b. Further studies are needed to investigate the pathways involved in NOX2-ROS regulation of B cell class switching.

### 4.3. NOX and NK Cells

Natural killer (NK) cells develop in the bone marrow or secondary lymphoid tissues from hematopoietic stem cell differentiation to common lymphoid progenitors, through a process regulated by non-hematopoietic stromal cell cytokine production. NK cells mature in the periphery, where they attain the ability to kill infected or cancerous cells [80,81]. NK cell cytolytic function is activated and regulated by receptors expressed on their surfaces. These receptors recognize altered proteins presented by infected or cancerous cells [82,83].

The role of NOX2 in NK cell maturation and function is understudied and existing research includes conflicting reports. NK cell numbers are reduced in peripheral blood from CGD patients [35], suggesting a role in NK cell development. NK cells from CGD patients have been reported with normal cytolytic function [84]. However, NK cell cytolytic function has been reported to require hydroxyl radical formation suggesting a role of NOX2 [85]. NOX2-ROS has been suggested to downmodulate NK cell function in mouse models of melanoma metastasis [86]. NK cells are sensitive to NOX2-ROS mediated apoptosis and functional inhibition [87,88,89]; therefore, malignant cells use NOX2-ROS as a means to escape NK cell immune surveillance. Malignant myelomonocytic and monocytic cells from patients with acute myeloid lymphoma express *CYBB* and produce large amount of ROS. The NOX2-ROS from these cells induce apoptosis of NK cells and even T cells through activation and a poly-[ADP-ribose] polymerase-1-dependent pathway [90]. Genetic ablation or chemical inhibition of NOX2 resulted in reduced distal metastasis of inoculated melanoma, while NK cell depletion abolished the inhibitory effect of NOX2 deficiency on tumor metastasis, and enhanced IFNγ production was observed from NOX2-deficient NK cells, suggesting NOX2-derived ROS facilitate metastasis of melanoma cells by downmodulating NK cell function [86]. In chronic myelogenous leukemia (CML), NK cell numbers and function are impaired [91]. Mellqvist et al. reported that NOX2-ROS from CML cells-induced apoptosis dysfunction of NK cells in a paracrine fashion [92]. When NOX2-ROS production was inhibited by idelalisib, a phosphatidylinositol-4,5-bisphosphate-3 kinase-δ (PI3Kδ) inhibitor, NK cells were rescued and tumor clearance improved in leukemia models [93]. NOX5 in oral tongue squamous cell carcinoma produce ROS to inhibit NK cytolytic function and this inhibition is thought to be mediated by CD271, a heterodimeric receptor that belongs to the tumor necrosis factor receptor superfamily on the surface of tumor cells [94]. In addition to ROS from tumor cells, NK cell cytolytic function is also inhibited by ROS generated from tumor-derived macrophages through down regulation of CD16ξ [95]. These studies all focused on the sensitivity of NK cells to exogenous NOX2-ROS. As noted above, NK cells express NOX. Similar to B cells, the major subtype of NOX expressed by NK cells is *CYBA* (Figure 2C). However, the role of NOX2-ROS from NK cells requires thorough investigation to identify the full impact of ROS in development, effector function, or inhibition of NK cells.

## 5. NOX and Dendritic Cells

Dendritic cells (DC) belong to antigen presenting cells. These cells can reside in tissue or stay in circulation. Dendritic cells engulf antigen, process antigen inside of the cells in phagosome and load processed antigen to MHC class II to present to CD4^+^ T cells, or load to MHC class I for cross-presentation to CD8^+^ T cells. Based on single cell RNA seq data, the major subtype of NOX expressed by dendritic cells is *CYBA* (Figure 2D). NOX are involved in antigen presentation restricted to both MHC class I and class II [96]. ROS are crucial for antigen presentation function. As a weak base, ROS alkalinize phagosome PH and affect proteolysis, which is essential for antigen processing and cross presentation to CD8^+^ T cells [97,98]. Other observations analyzing luminal microenvironment parameters within DC phagosomes indicated that NOX2 regulate proteolysis though redox modulation of local cysteine cathepsins, and is PH-independent, because phagosome acidification is unaffected [99]. Type 1 conventional DCs (cDC1s) are a subtype of DCs uniquely equipped to promote cytotoxic CD8^+^ T cell differentiation and are specialized against intracellular infections and tumors [100]. DNGR-1 (also known as CLEC9A), highly expressed by cDC1, is a receptor that binds dead cell debris and facilitates cross presentation of dead cell antigens [101]. The function of DNGR-1 requires a key tyrosine residue in its intracellular tail that allows the recruitment and activation of the tyrosine kinase SYK, which is also essential for cross presentation of dead cell antigens [101]. SYK can activate NADPH oxidase, leading to production of ROS that damage endosomal membranes and facilitate cross presentation [102]. In a mouse model, intact NOX2 in conventional dendritic cells (cDC) is necessary for DC to process and present antigen to CD4^+^ T cells in the initiation of EAE [103]. We have reported that dendritic cells from a mouse model deficient of NOX2 failed to cross-present antigen to CD8^+^ T cells; hence, the development of autoimmune Type 1 Diabetes was delayed in the NOD model [104]. However, NOX-ROS from DCs also regulate humoral immunity in a different way. In mouse models of streptococcus and listeria infection, DCs from mice carrying an *Ncf1* mutation showed higher infection-induced maturation markers CD80, CD86 and CD40, and higher MHC class II expression. These DCs from *Ncf1* null mice pulsed with bacterial antigen elicited higher specific antibody titers after adoptive transfer [105], suggesting a regulatory role of dendritic ROS on B cell antigen production.

## 6. Diseases Caused by NOX Deficiency

ROS production plays a major role in host defense against infectious pathogens and have been implicated in the development of autoimmunity. There are several human diseases caused by germline mutation in components of the NADPH oxidase system or defects in the enzymes responsible for ROS production.

**Figure 4:**
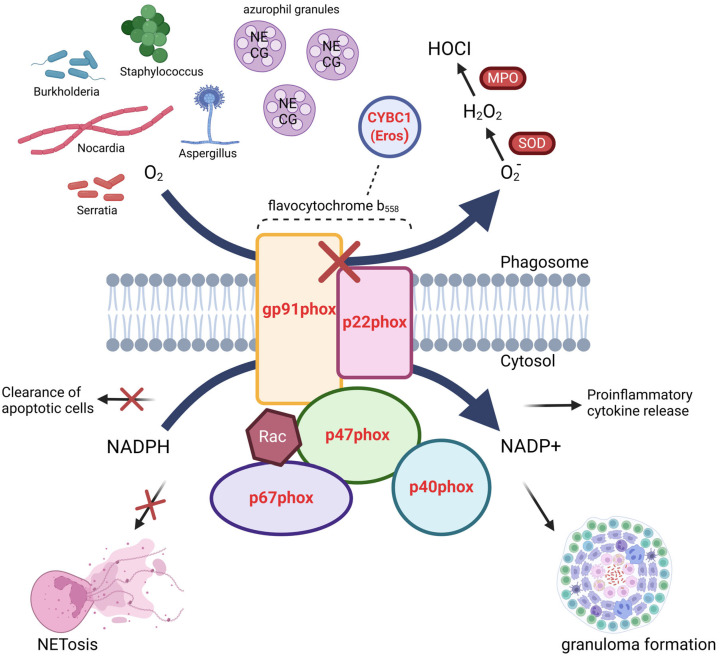
The NADPH oxidase complex and chronic granulomatous disease (CGD). Upon phagocyte activation, the various components of NADPH oxidase assemble to form the activated NADPH oxidase complex. Gp91phox shuttles electrons from cytosolic NADPH to molecular oxygen in the phagolysosome, resulting in the formation of superoxide ion (O_2_-). Superoxide ion is converted to hydrogen peroxide (H_2_O_2_) either spontaneously or by superoxide dismutase (SOD), and hydrogen peroxide is converted to hypochlorous acid (HOCl, bleach) by myeloperoxidase. These reactive oxygen species (ROS) are able to directly kill engulfed pathogens. In addition, azurophilc granules, which contain the anti-bacterial peptides neutrophil elastase (NE) and cathepsin G (CG), fuse with the phagolysosome during formation of the NADPH oxidase complex. The generation of ROS in the phagolysosome results in a rapid influx of K+ to counterbalance the negative charge, which in turn leads to the activation of the intraphagosomal peptides. The six proteins in which defects are known to result in CGD are shown in red, including five components of the NADPH oxidase complex and Eros, a chaperone protein essential for the expression of gp91phox. Defective ROS production results in infection with a subset of oxidase-positive bacteria and fungi (the five most common pathogens identified in CGD patients in the United States are shown here). Patients with CGD also have impaired clearance of apoptotic cells and formation of neutrophil extracellular traps (NETosis), both of which, in addition to other mechanisms not shown here, may contribute to granuloma formation and hyperinflammation.

Chronic granulomatous disease (CGD) is an inborn error of immunity caused by defects in the NADPH oxidase system that cause infection susceptibility and autoinflammatory disease (Figure 4). Defects in six genes responsible for NADPH oxidase assembly and production of ROS cause a similar clinical phenotype [106,107]. CGD occurs in approximately one in 200,000 live births in the United States but can occur more frequently in countries with founder populations or consanguinity [108,109].

As discussed in Section 2, the NADPH oxidase is comprised of six proteins pathogenic variants in *CYBB*, *CYBA*, *NCF1*, *NCF2*, *NCF4* cause CGD [110,111]. A sixth more recently described genetic cause of CGD is due to pathogenic variants in *CYBC1,* which encodes EROS. EROS is a chaperone protein required for the transport of the gp91phox/p22phox complex from the endoplasmic reticulum into the plasma membrane [112]. Pathogenic variants in *CYBB*, located on Xp21.1, are inherited in an X-linked manner and are the most common cause of CGD accounting for ~65–70% of cases [107]. Historically, patients with X-linked CGD fared poorly. Survival and infection susceptibility has now been shown to directly relate to the amount of residual superoxide produced by the NADPH oxidase [113]. Early missense mutations in *CYBB* (exception of His222) allow for some small amount of residual ROS production, whereas mutations beyond amino acid 310 contained within the NADPH, FAD, and the heme-binding domain often lead to absent protein expression [113]. Due to its location on the X-chromosome, large deletions that include *CYBB*, can include other closely located genes causing additional diseases. *KX*, which is <50 kB away from *CYBB*, encodes the Kell antigen; deletion of *KX* causes McLeod neurocanthocytosis syndrome, a multisystem disorder with CNS, cardiovascular, and hematologic manifestations [114]. Other genes in close proximity to *CYBB* are *RPGR* encoding retinitis pigmentosa GTPase regulator (telomeric) associated with RPGR-related retinitis pigmentosa; *DMD*, encoding dystrophin (telomeric) associated with Duchenne muscular dystrophy; and *OTC*, encoding ornithine transcarbamylase (centromeric) associated with ornithine transcarbamylase deficiency [115]. The second most common cause of CGD in the United States is due to pathogenic variants in *NCF1* occurring in ~25% of cases. Patients with p47^phox^-deficient CGD often have some small amount of residual ROS production. The majority of mutations described in *NCF1* are due to homozygous GT deletion.

This common deletion occurs due to the presence of two *NCF1* pseudogenes, each of which can carry the GT deletion. Recombination events lead to incorporation of the mutated pseudogene replacing non-mutated *NCF1*, causing truncation of functional *NCF1* [106]. *CYBA*, located on 16q24 encodes p22^phox^ and accounts for 7% of mutations causing CGD in the U.S. and is inherited in an autosomal recessive manner. p22^phox^ is the second subunit of cytochrome b_558_ and is required for gp91phox expression. Because of their interdependency, p22^phox^-deficient and gp91^phox^-deficient CGD are equal in severity. P67^phox^, encoded by *NCF2* located on 1q25.3, is a critical cytosolic factor for NADPH activation. P67^phox^ deficiency is inherited as autosomal recessive and accounts for ~15% of cases. Pathogenic variants in *NCF4*, encoding for p40^phox^, and *CYBC1* and *EROS* have been described in only a small number of individuals [112,116]. Interestingly, patients with p40^phox^-deficient CGD suffer from autoinflammatory disease, but are not at the same risk of invasive bacterial and fungal infections as are patients with other causes of CGD [117].

Diagnosis of CGD can be made by assessing direct measurement of neutrophil superoxide production. The Dihydrorhodamine (DHR) test is the most widely used assessment of neutrophil superoxide production and uses flow cytometry to measure the oxidation of dihydrorhodamine 123 to rhodamine 123 in PMA-stimulated neutrophils [118]. Pathogenic variants in the six genes causing CGD can be identified with next generation sequencing. Because *NCF1* is flanked by two pseudogenes, caution must be taken in interpretation to ensure that sequencing of the pseudogene is not mistaken for sequencing of *NCF1*. When large deletions are observed in *CYBB*, a chromosomal microarray is recommended to assess for contiguous gene rearrangement disorders. Susceptibility to serious invasive infections with a narrow spectrum of catalase positive bacteria and fungi is a hallmark of CGD. Catalase is an enzyme produced by certain bacteria and fungi and is responsible for catalyzing the breakdown of H_2_O_2_ into H_2_O and molecular oxygen. Catalase from catalase positive microbes is able to break down a small amount, if any, H_2_O_2_ produced by CGD neutrophils, effectively evading the host defense. The most common pathogens to cause infection in patients with CGD in the U.S. are *Staphylococcal aureus*, *Burkholderia cepacia* complex, *Serratia marcescens*, *Nocardia* sp., and *Aspergillus* sp. In less developed countries or in parts of the world where tuberculosis is endemic or vaccinated against, Salmonella species, *Mycobacterium* tuberculosis, and Bacille Calmette–Guerin can cause infection. The most common sites of infection are the skin, lymph nodes, liver, lungs, and blood [119,120,121]. Inflammatory complications are another common manifestation of CGD [122]. Gastrointestinal complications occur in about 50% of cases with inflammatory bowel disease similar to Crohn’s disease being the most common [123,124,125,126]. Symptoms of CGD-IBD include failure to thrive, abdominal pain, iron deficiency anemia, diarrhea, hematochezia, and stooling with urgency, frequency, and tenesmus. Treatment of CGD-IBD is challenging and relies heavily on use of corticosteroids. Despite their success, fatal infections have occurred in patients with CGD-IBD treated with TNFα inhibitors [127,128]. Variable success has been reported with the use of anakinra, vedolizumab or stekinumab [123,124,125,126]. Inflammatory disease of the liver includes non-regenerative hyperplasia, portal hypertension, and chronic transaminase elevation. Granulomatous and interstitial disease of the lung can cause hypoxemia and pulmonary hypertension [129,130,131], as reviewed in [122]. Lastly, patients with CGD are plagued with organ specific and systemic autoimmunity, including systemic lupus erythematosus (SLE), rheumatoid arthritis (RA), antiphospholipid syndrome, IgA nephropathy, autoimmune cytopenias [122,132]. An exaggerated inflammatory response to infection is also observed in patients with CGD. Treatment of Nocardia pneumonia [133] and Stapahylococcus aureus liver abscesses [134,135] often require concurrent treatment with corticosteroids in addition to anti-microbials in order to blunt the local and systemic inflammatory response. Hyperinflammation in response to infection and autoimmunity can be severe enough to trigger hemophagocytic lymphohistiocytosis (HLH) [136,137]. HLH in CGD has a high mortality rate and requires a high index of suspicion for recognition and quick initiation of immunosuppressive therapy. The biologic mechanism of the development of inflammatory disease and autoimmunity in CGD is poorly understood. Several pathways have been explored and proposed, but the exact mechanism is likely multifactorial. Residual ROS production correlates with susceptibility to infections but has no association for the development of inflammatory disease [113]. Further, female carriers of X-linked CGD, in which one X chromosome carries the normal functioning allele, while the second X chromosome harbors the mutant *CYBB,* are afflicted with high rates of autoimmunity and inflammatory disease, similar to their CGD counterparts [138,139,140]. Taken together, these data indicate that a normal amount of ROS is necessary to prevent development of inflammatory disease in CGD and CGD carriers but the amount of reduction below normal does not correlate with symptom susceptibility or onset.

Prognosis of patients with CGD has improved substantially over the last 50 years with >80% living into the fifth decade of life, but severity of the disease and overall prognosis are predicted by genotype and residual superoxide generated [113]. Treatment of CGD relies on lifelong surveillance of infections, autoimmunity, and inflammatory disease and use of lifelong prophylaxis with anti-microbials and immune modulation with IFNγ [141,142]. Corticosteroids are the backbone to treatment of inflammatory disease. Treatment of CGD-IBD has also had variable success with use of biologics, including vedolizumab, anakinra, and ustekinumab (reviewed in [122]). Definitive treatment can be successful with a hematopoietic cell transplant [143,144] and is recommended for most patients. Alternative therapies, including gene therapy, are being investigated [145,146].

## 7. Myeloperoxidase Deficiency

Myeloperoxidase (MPO) is the heme-containing enzyme necessary for the conversion of H_2_O_2_ to hypochlorous acid (HOCL) after NADPH activation. MPO is expressed in early myeloid progenitors within primary azurophilic granules. MPO deficiency is common, occurring in approximately 1 in 2000–4000, but is not commonly associated with enhanced susceptibility to infection. Infection with candida and paracoccidiomycosis have been reported, but occur in the setting of other infection pre-disposing morbidities [147,148].

## 8. Glucose-6-Phosphate Dehydrogenase Deficiency

G6PD is the rate-limiting enzyme catalyzing the conversion of glucose-6 phosphate into 6-phosphogluconolactone as the first step in the pentose phosphate pathway and leading to the production of NADPH. Inheritance of G6PD deficiency occurs in an X-linked dominant pattern. G6PD deficiency is one of the most common enzymopathies affecting ~400 million people worldwide [149]. The pentose phosphate pathway is the primary source of NADPH in red blood cells, causing red blood cells to become vulnerable to oxidative stress. G6PD deficiency can be mild to severe with five different classes of variants. In the setting of oxidative stress, red blood cells hemolyze and cause anemia. Severe loss of G6PD enzyme activity can cause severe NADPH oxidase deficiency and subsequent reduced ROS activity in phagocytes. ROS activity as measured by DHR can be impaired, and a CGD-like clinical phenotype can be observed [150,151].

## 9. NOX Deficiency and Autoimmunity

Historically, ROS produced by NOX2 have been implicated in the development of autoimmunity and considered harmful mediators of inflammation [152]. More recently, a growing body of data has shown that ROS also have anti-inflammatory effects, preventing autoimmune disease and that there is a fine balance between quantity, timing, and location of ROS production that tips their effect to helpful versus harmful (reviewed in [153,154]). In animal models, *Ncf1* mutation promotes the development of RA, SLE, and Sjogren’s syndrome [155,156,157,158]. Disease pathogenesis mechanisms include NET formation, activation of autoreactive T cells, and enhanced type I interferon responses [37,159,160,161]. CGD is one example in which ROS deficiency leads not only to infection susceptibility but to both organ specific and systemic autoimmunity [122,132] and, interestingly, many of the mechanisms described in the development of autoimmunity are the same or similar to that described as potential mechanisms of inflammatory disease pathogenesis in humans with CGD.

### 9.1. Rheumatoid Arthritis

Rheumatoid arthritis (RA) is an inflammatory arthritis characterized by joint inflammation and destruction with childhood or adult onset [162]. In ongoing arthritic inflammation, circulating monocytes and neutrophils have increased NOX2 activity and ROS production is high in neutrophils found in synovial fluid from inflamed joints [163,164]. Further, chrondrocytes are stimulated to undergo apoptosis in a high ROS environment, thereby preventing normal cartilage function and joint repair [165]. Contrary to their role in propagating joint inflammation, genetic alteration in genes encoding the NADPH oxidase have shown protective effects against the development of RA. Recovery of ROS in an *Ncf1* polymorphism model in rats has a protective effect from the development of RA [155]. More severe arthritis, earlier onset, enhanced cartilage destruction, and higher levels of anti-collagen antibodies have also been observed in a collagen-induced arthritis *Ncf1* mutated mouse model [77]. In humans, polymorphisms in *NCF4*, *NCF2*, *RAC2* are associated with increased RA incidence [166], while increased copy number of *NCF1* is a protective factor against the development of RA [157].

### 9.2. Systemic Lupus Erythematosus (SLE)

SLE is a chronic autoimmune disease characterized by inflammation of the joints, kidneys, skin, and lungs; autoantibodies directed against nuclear components are hallmark of the disease [167]. Chronic inflammation in SLE is associated with heightened ROS, which triggers oxidative stress, DNA damage and endothelial and epithelial cell necrosis [168]. This cascade leads to T cell and B cell self-reactivity. In contrast, several mouse models have shown the harmful effects on ROS deficiency causing development of SLE or a worsened SLE phenotype. Mice with *Ncf1* mutation and low ROS develop elevated levels of lupus associated antibodies, IgG and C3 deposition in glomeruli, and heightened expression of IFNg-stimulated genes [37,77]. Crossing MRL.Fas^lpr^ with NOX2-deficient mice results in increased production of anti-Smith and anti-RNA antibodies, worsened renal disease, and increased spleen weight all consistent with worsened SLE disease [169]. In humans, SNPs in *NCF2* are strongly associated with SLE risk but in childhood and adult onset SLE [170].

### 9.3. Psoriasis

Psoriasis is a chronic inflammatory disease of the skin that can progress in some to cause arthritis with joint destruction [171]. Numerous studies have shown an association with oxidative stress and development of psoriasis or psoriatic arthritis [172,173,174]. However, ROS have also shown protective effects by increasing T reg function and enhancing expression of indoleamine 2,3-dioxygenase. Treatments employed to treat psoriasis, including hyperbaric oxygen, imiquimod, and phototherapy, have shown associated increases in ROS while providing a clinical benefit, further agreeing with the protective effects that ROS may also provide [175,176].

## 10. Treatment

Activators or agonists of NOX2 have the potential to be therapeutic in a number of autoimmune and inflammatory conditions, including those detailed above (see detailed review [177]). Despite their obvious role in causing and propagating autoimmune and inflammatory diseases, there are no FDA approved therapies that act to enhance NOX2. As mentioned in Section 6, treatment of CGD depends on prevention of infections and surveillance of autoinflammation. No specific pharmacologic therapies enhance NOX2 production. Definitive therapy with HSCT and gene therapy restore NADPH oxidase function within the hematopoietic compartment and are curative at treating and preventing disease related sequelae. Gene therapy is still under clinical investigation and is not yet approved in the U.S. for treatment of CGD [146,178].

Direct engagement of integrin receptors, Fc-receptors, C-type lectin receptors, or G-protein coupled receptors, such as the formyl peptide receptor (FPR) family initiate assembly of the NADPH complex, and are being investigated to exploit to enhance NOX2 production. In particular, FPR agonists have demonstrated significant anti-inflammatory effects and are an active area of investigation [179]. Screening campaigns of small molecules that induce NOX2 production have identified several novel targets [177].

## 11. Summary and Gaps in the Knowledge of the Field

NOX-derived ROS play important roles as both effector molecules and signaling mediators. The role of ROS in each immune cell type is distinct yet not completely independent. To date, no studies explain the role of NOX2-ROS on T cell differentiation and signaling. The interplay between immune cells is a key factor in maintaining immune balance. NOX-ROS is a crucial mediator of these interplays. NOX-ROS from accessory cells, i.e., macrophages and dendritic cells, regulate the activity of T cells and B cells. Proper amount of ROS production is key to maintaining physiological balance. Controversial results exist concerning the role of ROS in the development of autoimmunity, mostly due to different disease entity, ROS from different cell types or if ROS production is completely inhibited in these studies. Because NOX deficiency directly results in the disease CGD, and the overt clinical manifestation is uncontrollable infections, most research in the field focuses on roles of NOX-ROS in neutrophils and macrophages. Figure 4 shows the function of NOX2 and pathways involved in NOX deficiency-caused CGD. Further investigation is needed to understand how NOX-ROS from T cells, B cells and NK cells per se participate in immune regulation, detail signaling pathways involved in the regulation, and provide novel insight into prevention as well as treatment of immune deficiency and autoimmunity.
